# Acute Inositol-Stabilized Arginine Silicate Improves Cognitive Outcomes in Healthy Adults

**DOI:** 10.3390/nu13124272

**Published:** 2021-11-26

**Authors:** Joshua L. Gills, Anthony Campitelli, Megan Jones, Sally Paulson, Jennifer Rae Myers, Erica N. Madero, Jordan M. Glenn, James Komorowski, Michelle Gray

**Affiliations:** 1 Department of Health, Human Performance and Recreation, Exercise Science Research Center, University of Arkansas, Fayetteville, AR 72701, USA; jgills@uark.edu (J.L.G.); amcampit@uark.edu (A.C.); mdl016@uark.edu (M.J.); 2 St. Elizabeth Healthcare, Edgewood, KY 41017, USA; paulsonsa08@gmail.com; 3 Neurotrack Technologies, Inc., Redwood City, CA 94063, USA; jennifer@neurotrack.com (J.R.M.); erica@neurotrack.com (E.N.M.); Jordan.mckenzie.glenn@gmail.com (J.M.G.); 4 JDS Therapeutics LLC., Harrison, NY 10528, USA; jkomorowski@jdstherapeutics.com

**Keywords:** inositol-stabilized arginine silicate, cognition, nitric oxide

## Abstract

Inositol-stabilized arginine silicate (ASI) is an ergogenic aid that upregulates nitric oxide. Acute ASI supplementation improves working memory and processing speed in young adults but there is a lack of data examining other cognitive tasks. Therefore, the purpose of this study was to examine acute ASI effects on young healthy adults by assessing multiple cognitive domains. Nineteen young adults (20.9 ± 3.2 years) completed this randomized, double-blind, crossover study consuming ASI (1.5 g ASI + 12 g dextrose) and placebo (12 g dextrose). The participants completed the Repeatable Battery for the Assessment of Neuropsychological Status (RBANS) and two digital cognitive assessments before consuming the supplement and then completed the same battery of tests 60 min post-supplementation. Repeated measures ANOVA demonstrated that ASI consumption significantly improved total RBANS and immediate memory scores compared to the placebo (*p* < 0.05). However, no significant differences were displayed between trials for other cognitive domains (*p* > 0.05). Acute ASI ingestion increased overall RBANS scores and immediate memory scores in young adults. More research is needed to examine the acute effects of ASI on other domains of cognition, in older populations, and its long-term effects on cognition.

## 1. Introduction

The sports nutrition industry is a growing, lucrative market; however, a common pitfall within the industry is a lack of scientific efficacy with many over-the-counter supplements. Inositol-stabilized arginine silicate (ASI: Nitrosigine^®^, Nutrition 21 LLC, Harrison, NY) is an ergogenic aid that is gaining more scientific backing due to its ability to significantly improve the bioavailability and absorption of arginine and silicon, thus increasing nitric oxide (NO) circulation [1]. Studies have shown that a single dose of ASI significantly enhances plasma arginine levels up to six hours after consumption [2]. Enhanced NO availability, via arginine administration, induces vasodilation, increases blood flow to the working musculature, improves cardiovascular health, lowers blood pressure, improves exercise performance, and enhances memory [3,4]. Moreover, research demonstrates that NO may be a prospective therapeutic approach in managing and mitigating mild cognitive impairment due to its mechanistic action, increasing systemic blood flow and, conceivably, cerebral circulation [5,6]. Among individuals with learning and memory impairments, the beneficial effects of NO in improving these impairments are well-known [7]. Furthermore, enhancing learning and memory function could potentially delay or slow the cognitive decline process. While it has been established that augmented NO levels improve cognitive functioning in impaired individuals, there have also been studies demonstrating beneficial effects in cognitively normal individuals. In healthy adults, arginine supplementation demonstrated decreases in participants’ stress and anxiety levels [8]. In recent investigations, ASI ingestion augmented mental concentration and sharpness potentially through augmented NO levels, which enhanced blood circulation and aided in the distribution of nutrients to the brain [1,3].

Silicon is a trace element, found in plant foods, thought to increase the bioavailability of L-arginine [9], and plays an essential role in the body (e.g., skin, hair, immune function) [10]. Studies have shown that silica potentially helps to maintain vascular integrity in vascular diseases that occur with age [11]. Additionally, evidence demonstrates a link between silica intake and a decreased likelihood of Alzheimer’s disease and other forms of dementia, possibly due to the mechanistic action of silica binding to and eliminating aluminum from the brain, indicating a positive association between silica ingestion and brain health [12]. While slowing cognitive decline is important in aging, it is also imperative to maintain or enhance cognitive capacity in young adults, which may result in better performance in athletic, academic, and occupational settings.

As such, silicon and arginine combined have shown promising results for improving cognitive function in young adults. ASI supplementation has led to increases in processing speed of up to 45% while completing the Trail Making Test (TMT) [3]. Moreover, ASI ingestion has been shown to increase processing speed after a strenuous bout of exercise compared to placebo [1]. In both of these studies, the TMT was used to assess processing speed. While this test is valuable, it only measures a limited scope of cognitive processes. Therefore, the purpose of this investigation was to evaluate the acute effects of ASI supplementation in young healthy adults on multiple domains of cognition. We hypothesized that acute ASI ingestion should upregulate NO, thereby increasing performance on cognitive tasks in healthy adults. We believed that acute ASI consumption would increase (a) cognitive performance from pre-supplementation to post-supplementation and (b) produce better cognitive battery scores than a placebo (PLA) trial.

## 2. Materials and Methods

### 2.1. Participants

This protocol was a double-blind, crossover, placebo-controlled study. The overall sample was 63% female (*n* = 12 participants) and the mean age of the participants was 21.0 ± 3.2 years (range 18–28 years). Of the 19 participants enrolled in the study, 16 completed both trials. Individuals were excluded from the study if they had disabling vision loss; were unable to complete the calibration procedure for the web camera; had a history of substance abuse, learning disability, neurological illness (i.e., stroke, tumor), or psychiatric illness; or if they consumed a commercial pre-workout product that included arginine or ASI on the ingredients list. All the participants were required to sign a statement of informed consent approved by University of Arkansas’ Institutional Review Board (Protocol #: 1909220945R001).

### 2.2. Procedures

**Figure 1:**
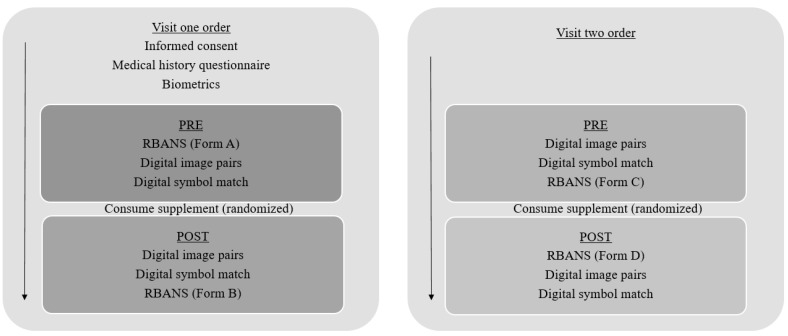
Trial order.

The participants reported to the Exercise Science Research Center at the University of Arkansas on two separate occasions. The first visit included the completion of an informed consent document, a medical history questionnaire, and biometric assessments upon arrival on the first trial. The participants completed three different cognitive tests (PRE), consumed ASI (1.5 g and 8 g of dextrose) or PLA (8 g of dextrose), and observed a 60 min digestion period; three different versions of the same cognitive tests were administered for both trials (POST) (Figure 1). The second testing session occurred at least 7 days later (Figure 1). The test order was randomized for each visit. The protocol (Protocol #: 1909220945R001) was approved following Full Board Review by the IRB Committee that oversees research with human subjects.

### 2.3. Biometric Assessments

Then biometric assessments included height, weight, and body composition. Height was assessed using a standing stadiometer (SECA; Hamburg, Deutschland, Germany). During this assessment, the subjects were asked to remove their shoes and stand as straight as possible. Height was recorded to the nearest 0.1 cm. Weight was measured using a balance-beam scale (Sunbeam Products, Inc., McCook, IL, USA); the participants were asked to remove their shoes and any heavy clothing (sweaters, jackets/coats), and empty their pockets. Weight was measured to the nearest 0.1 kg. Body composition was measured using dual-energy X-ray absorptiometry (DXA; General Electric Company, Madison, WI, USA).

### 2.4. Cognitive Assessments

The cognitive assessments were completed via computer and paper-pencil mediums during both visits.

#### 2.4.1. Repeatable Battery for the Assessment of Neuropsychological Status (RBANS)

Each participant was individually administered the RBANS assessment (RBANS; Forms A, B, C, & D). The RBANS assessment was completed on an iPad along with paper and pencil. The RBANS assessment construction is explained in detail elsewhere [13]. Briefly, the RBANS is made up of 12 subtests that are used to calculate five index scores and a total score. Test catalogues included: Immediate memory (list learning and story memory tasks), visuospatial/constructional (comprised of figure copy and line orientation tasks), language (picture naming and semantic fluency tasks), attention (digit span and coding tasks), and delayed memory (list recall, story recall, figure recall, and list recognition tasks). Each index score falls within an age-adjusted score [13]. The index scores are combined to produce a total score, which is a summary score of the participant’s performance on the RBANS. The RBANS test takes ~30 min to administer and finish. Minimal clinically important differences (MCID) exist for total RBANS scores (≥5.8 for within-group, ≥3.3 between-group), immediate memory scores (≥6.2 for within group, ≥4.1 between group), delayed memory scores (≥5.4 for within group, ≥3.7 between group) and language scores (≥5.7 for within group, ≥4.8 between group). Previous research showed that the RBANS is significantly correlated with more extensive exams, such as the Wechsler Adult Intelligence Scale III and the Wechsler Memory Scale III; it also offers strong test-retest reliability [14,15].

#### 2.4.2. Digital Image Pairs

Image Pairs is an eye-tracking-based task measuring visual recognition memory and learning [16,17,18]. The visual paired comparison portion of the test measures the participant’s ability to recognize images already viewed during the familiarization phase. The paired recognition portion of the test measures the participant’s ability to learn and identify image pairs they have been tasked with learning.

There are four phases (one familiarization phase, one learning phase, and two testing phases) for this specific exam. During the first (familiarization) phase, the participants were presented with 10 pairs of identical visual images for 5 s. After the first phase of the VPC, a continuous and cumulative delay occurred across each test trial. Before the second (testing) phase began, the participants were instructed to look at the novel or unfamiliar image. During the test phase, the participants were presented with 10 pairs of images, including one from the familiarization phase and one novel image. The amount of time a participant spent viewing the novel image relative to the total viewing time created a novelty preference score, with higher scores indicating better declarative memory and lower scores suggesting impaired cognitive function [16]. During phase three (learning), the participants were shown the same 10 paired images from phase 2 and asked to remember the paired images for phase four. During phase four (testing), the participants were presented with 25 image pairs. Ten of the pairs were from phase three (correct), ten contained familiar images that were paired incorrectly (incorrect), and five contained sham images that had never been viewed before (sham). For each image pair, the participants selected either yes or no for correct pairs, incorrect pairs, or sham pairs. Accuracy scores were produced from phase 4. Eye movements were tracked and scored. Detailed scoring information is published elsewhere [16]. Previous research has found that digital image pairs hhave significant associations with the Montreal Cognitive Assessment (MoCA) and digit coding symbol test [16,17,18] while offering strong test-retest reliability [17,18].

#### 2.4.3. Digital Symbol Match

Symbol Match is a processing speed and executive functioning task that utilizes a paired verification or rejection paradigm (forced choice). The participants were instructed to determine whether two symbols are equal or unequal utilizing a legend with nine number/symbol pairs over a two minute time span. At the conclusion of the task, a brief implicit learning trial was administered without the legend present. Scoring was determined by speed and the number of correct trials subtracted by the number of incorrect trials, producing a derived accuracy score for the learning trial. Previous investigations identified temporal stability and good test-retest reliability during this task [19].

### 2.5. Nutritional Data

The participants were asked to keep track of their dietary intake 24 h before each trial. The participants recorded 24 h dietary recall upon arrival of the visit. The macronutrient and total caloric values were calculated based on publicly available nutrient profiles published by the USDA. The nutritional data were assessed using nutritionist pro (Axxya Systems; Redmond, WA, USA).

## 3. Statistical Analysis

The Statistical Package for the Social Sciences (SPSS, version 25) (Armonk, NY, USA) was used to conduct all the analyses. The normal distribution of the data was assessed using histograms and boxplots. Descriptive statistics (means and standard deviations) were calculated for all the data. An a priori power analysis based on 0.80 for power, alpha of 0.05, and two-tailed while using a repeat measures ANOVA (RMANOVA) suggested that 12 participants was a sufficient number with which to obtain meaningful results. Partial eta-squared (η^2^_p_) was used to demonstrate the effect size. The following criteria for η^2^_p_ were used to explain the practical significance of the findings: small (0.01), moderate (0.06), and large (0.14).

An RMANOVA was utilized to analyze within-trial (PRE to POST supplement) differences for both supplement trials. The pre-supplement scores for ASI and PLA supplement trials were covariates for between-trial analyses. The percentage changes from the pre-supplement and post-supplement trials were calculated for both supplements. Dependent *t*-tests were used to examine differences between pre-trial nutritional intakes between both supplement trials. Statistical outliers were determined through box and whisker plots. No statistical outliers were found as no data points were beyond 3three times the interquartile range. Statistical significance was set at α = 0.05. The demographic information is presented as means ± SD and the inferential data are presented as means ± SE.

## 4. Results

### 4.1. RBANS

**Figure 2:**
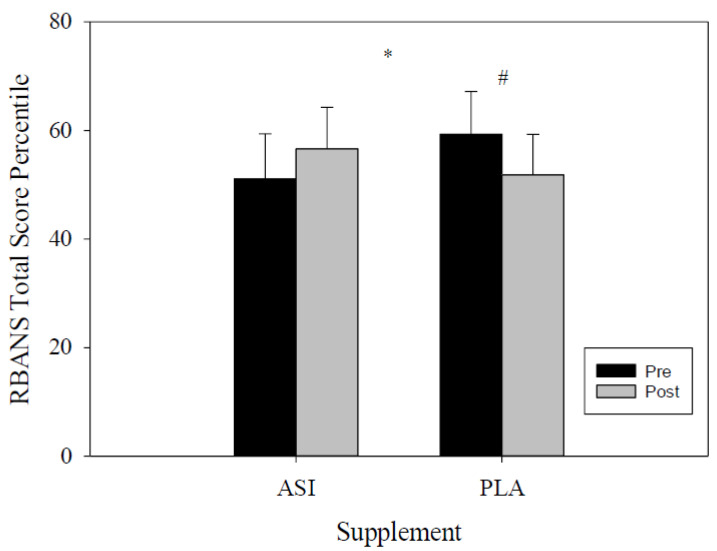
Total RBANS percentile score. * indicates differences between supplement (*p* < 0.05); #—indicates differences within-trial.

The total RBANS percentile scores were analyzed through RMANOVA for both PLA and ASI trials. The within-trial analysis differences showed no significant differences from pre- to post-supplement for both trials (*F*(1, 27) = 0.331, *p* = 0.57; η^2^*_p_ =* 0.01). However, a group by time effect was identified between trials (*F*(1, 27) = 4.379, *p* = 0.04; η^2^*_p_* = 0.14; Figure 2) with an increase in performance for ASI scores and a decrease in PLA scores from pre- to post-trial (Table 1). The between-trial differences showed a significant increase in total RBANS scores during the ASI trial compared to the PLA trial (*F*(1, 30) = 5.658, *p* = 0.02; η^2^*_p_ =* 0.18; Table 1). ASI supplementation exhibited an 11% increase in total RBANS score.

**Figure 3:**
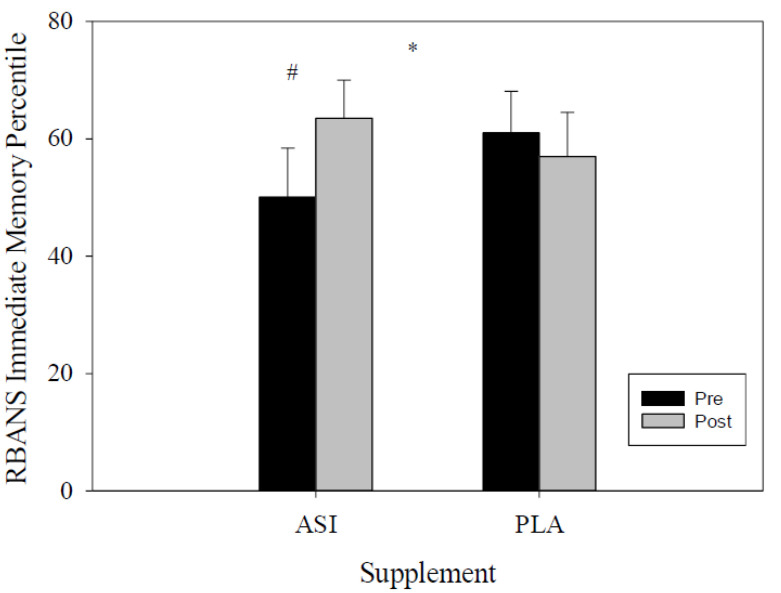
Immediate memory percentile score. * indicates differences between supplement; # indicates differences within-trial.

The immediate memory percentile scores were examined through RMANOVA for both the PLA and ASI trials. There were no significant differences within-trial (pre-to-post supplement) (*F*(1, 27) = 1.098, *p* = 0.30; η^2^*_p_* = 0.04). A group by time effect was noticed between trials (*F*(1, 27) = 6.213, *p* = 0.01; η^2^*_p_* = 0.19; Figure 3), with an enhanced performance observed during ASI supplementation and a decreased performance after the PLA trial (Table 1; Table 2). Moreover, ASI showed a significant increase in scores compared to the PLA trial (*F*(1, 31) = 4.292, *p* = 0.04; η^2^*_p_* = 0.12; Table 1); the ASI trial exhibited a 27% increase from pre- to post-supplementation scores.

**Figure 4:**
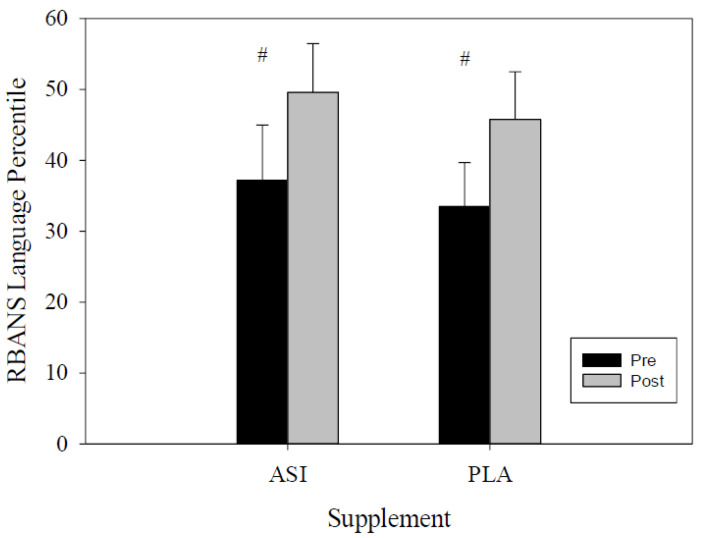
Language percentile score. #-indicates differences within-trial.

The language percentile scores were evaluated through RMANOVA for both the PLA and ASI trials. There was a significant increase in the scores exhibited within-trial (pre-post supplement scores; *F*(1, 27) = 7.483, *p* = 0.01; η^2^*_p_* = 0.22). However, no significant differences were identified between supplement trials (*F*(1, 32) = 2.195, *p* = 0.84; η^2^*_p_* = 0.06; Figure 4).

**Figure 5:**
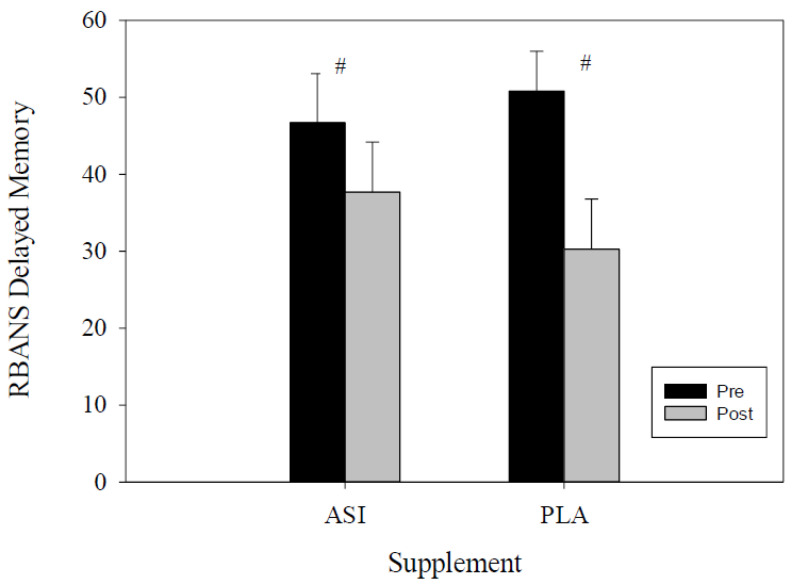
Delayed memory percentile score. # indicates differences within-trial.

The delayed memory percentile scores were examined through RMANOVA. Significant within-trial differences were found (*F*(1, 27) = 19.119, *p* < 0.001; η^2^*_p_* = 0.42; Figure 5). No significant between-trial differences were found between supplement trials (*F*(1, 30) = 2.109, *p* = 0.15; η^2^*_p_* = 0.06; Table 1).

No significant differences were identified during the attention tasks within or between supplement trials (*p* > 0.05; Table 1). Visuospatial/constructional abilities did not differ between trials during the placebo trial (*p* > 0.05; Table 1).

### 4.2. Digital Image Pairs and Symbol Match

The image pairs percentile scores displayed within-trial differences (*F*(1, 27) = 11.002, *p* = 0.003; η^2^*_p_* = 0.05). No significant differences were found between the ASI trial and the PLA trial (*F*(1, 29) = 0.513, *p* > 0.05; Table 2).

Moreover, the digital symbol match percentile scores showed no within-trial or between-trial differences (*p* > 0.05; Table 2). The ASI trial exhibited a 6% increase from pre- to post-supplementation scores while the PLA trial showed a 1% increase from pre- to post-supplement scores.

### 4.3. Nutritional Analysis

A dependent *t*-test was used to analyze the differences in caloric (kcal), protein (g), carbohydrate (g), and total fat (g) intake variables between trials. There were no significant differences in calorie, carbohydrate, protein, or total fat intake in the 24 h prior to each trial (*p* > 0.05)

## 5. Discussion

The primary aims of this study were to examine ASI’s ability to improve cognitive battery performance in healthy participants. Our a priori hypothesis predicted that ASI supplementation would augment various cognitive domains compared to PLA. Our hypotheses were partially supported; acute ASI ingestion improved global cognition and immediate memory performance compared to the PLA. In addition, ASI significantly improved immediate memory scores by 27%. However, there were no significant differences between supplement trials during other cognitive tasks. However, MCID improved in terms of RBANS total score and immediate and delayed memory outcomes during the ASI supplementation trials compared to the PLA trials, suggesting acute beneficial acute after ASI ingestion.

Our results align with previous ASI findings, in which mental focus and acuity were obtained through the increase in NO levels [1,3]. Improved NO levels potentially enhanced blood flow to the brain, providing increased glucose uptake, but this mechanism cannot be confirmed through the current study. However, previous studies have demonstrated that acute ASI supplementation increases blood flow in the brachial artery and augments plasma arginine levels for up to 6 h [2,20]. Moreover, immediate memory improved significantly for the ASI trial group compared to the PLA group. Previous investigations have indicated that NO upregulation augments memory and learning tasks [7]. ASI improved RBANS total and immediate memory scores, while preserving delayed memory scores compared to the PLA trial. The delayed memory scores decreased across both trials, but to a greater degree during the PLA trial. The length of the trial (~3-h) may have contributed to the reduction in performance across trials. The length of the total trial in combination with the 2 h fast pre-trial may have caused participant fatigue during the delayed memory portion of post-supplement testing.

Language scores improved during both supplement trials. However, the only significant improvement was shown in the picture naming assessment during the PLA trial. However, the participants were able to improve by one additional point for both trials from pre- to post-supplementation (Table 2). Processing speed (attention domain and digit symbol match) did not change significantly during the ASI trial. Although previous investigations have identified a 45% increase in processing speed after ASI consumption [1,3], our study did not demonstrate this outcome using digit symbol coding tasks on paper or electronically. Kalman et al. (2016) [3] utilized the TMT, which potentially caused the discrepancy among outcomes. The TMT is known to have a significant learning effect when completing multiple trials [21]. However, the current investigation found that total RBANS scores were significantly different between supplement trials. Therefore, global cognitive functioning was improved with the supplementation of ASI in young adults.

The design of the current investigation as a randomized, double-blind, placebo-controlled study is a strength because it removes common sources of bias in less rigorous study designs. The limitations of this investigation include missing data from participants that did not complete both trials and unequal numbers of subjects between sex. Sex differences in cognition are usually more prevalent in early stages of life, but it could be of interest to compare differences between males and females consuming ASI [22]. Next, the study design took three hours per visit; the participants could not eat or drink anything before the trial and were required to wait two hours after the trial began to start the post-supplementation tests. This may have impacted their performance. As seen in the results section, post-supplementation delayed memory scores decreased during the RBANS assessment. Lastly, the small sample size limits the generalizations that can be taken from this investigation.

The data from this investigation demonstrated the clinical relevance of ASI for healthy populations. ASI supplementation increased overall performance on RBANS and significantly improved memory compared to the PLA trial. One converse finding was the lack of difference in processing speed, which was seen in previous ASI supplementation studies [1,3]. Further studies should examine how much ASI supplementation can improve cognition scores in older populations and explore sex differences. Furthermore, future studies should decrease the amount of time participants must spend in the laboratory to account for fatigue as a confounding variable.

In conclusion, the results of this investigation demonstrate that acute ASI supplementation increased overall cognitive scores in healthy participants. Specifically, memory scores significantly increased in ASI vs. PLA. No significant differences were identified between groups for attention, processing speed, recognition memory, language, and visuospatial skills. However, it should be noted that a minimal clinically meaningful difference was observed for immediate memory, delayed memory, and global cognition. Thus, acute ASI supplementation improved overall cognitive function in healthy adults.

## Figures and Tables

**Table 1:** RBANS data.

Variable	Supplement	Pre	Post	Lower CI	Upper CI	*p*-Value
Total Score Index	ASI	102.7 ± 4.8	105.5 ± 4.6	−6.67	1.14	0.017 *
	PLA	104.9 ± 3.8	100.9 ± 3.5	−0.10	8.22	
Total Score Percentile	ASI	51.1 ± 8.3	56.6 ± 7.6	−13.71	2.70	0.024 *
	PLA	59.3 ± 7.9	51.8 ± 7.5	0.35	14.65	
Immediate Memory Index	ASI	101.8 ± 4.7	107.2 ± 3.7	−11.44	0.61	0.108
	PLA	105.8 ± 4.1	103.5 ± 3.9	−4.69	9.16	
Immediate Memory Percentile	ASI	50.1 ± 8.3	63.5 ± 6.5	−23.45	−3.4	0.047 *
	PLA	59.3 ± 7.9	51.8 ± 7.5	−8.04	15.9	
Visuospatial/Constructional Index	ASI	107.5 ± 3.1	106.3 ± 4.1	−4.79	7.15	0.270
	PLA	110.9 ± 4.3	105.0 ± 3.6	0.98	10.79	
Visuospatial/Constructional Percentile	ASI	63.9 ± 6.3	61.9 ± 8.1	−10.39	14.52	0.186
	PLA	75.4 ± 7.4	61.2 ± 6.7	5.15	23.32	
Language Index	ASI	90.2 ± 4.9	99.6 ± 3.2	−15.71	−3.12	0.689
	PLA	90.1 ± 3.8	98.2 ± 3.1	−15.88	−0.24	
Language Percentile	ASI	37.2 ± 7.8	49.6 ± 6.9	−23.14	−1.69	0.148
	PLA	33.5 ± 6.2	45.8 ± 6.7	−26.24	1.58	
Attention Index	ASI	109.1 ± 4.1	110.5 ± 5.2	−8.59	5.89	0.521
	PLA	109.0 ± 3.7	107.8 ± 3.6	−3.13	5.60	
Attention Percentile	ASI	63.4 ± 6.9	63.4 ± 7.7	−11.29	11.19	0.960
	PLA	66.6 ± 6.7	66.6 ± 7.5	−7.11	6.96	
Delayed Memory Index	ASI	98.8 ± 3.1	92.8 ± 3.5	0.42	11.70	0.272
	PLA	100.4 ± 2.3	89.4 ± 3.6	4.32	17.55	
Delayed Memory Percentile	ASI	46.7 ± 6.4	37.7 ± 6.5	−1.23	19.11	0.157
	PLA	50.8 ± 5.2	30.3 ± 6.5	8.43	32.57	

Note. means ± SE, ASI: Bonded Arginine Silicate, PLA: Placebo, CI = 95% confidence interval of the mean difference, * *p*-value < 0.05 for between-differences.

**Table 2:** RBANS subtest raw scores and Digital cognitive test data.

Variable	Supplement	Pre	Post	Lower CI	Upper CI	*p*-Value
List Learning (IM)	ASI	29.9 ± 1.2	31.7 ± 1.1	−4.41	0.81	0.169
	PLA	31.7 ± 1.0	30.6 ± 0.9	−1.06	3.18	
Story Memory (IM)	ASI	18.5 ± 1.0	19.7 ± 0.9	−2.99	0.73	0.476
	PLA	19.4 ± 1.0	19.4 ± 1.0	−1.59	1.38	
Figure Copy (VSC)	ASI	19.7 ± 0.2	19.6 ± 0.3	−0.32	0.46	0.308
	PLA	19.4 ± 1.0	19.4 ± 0.7	−0.13	0.83	
Line Orientation (VSC)	ASI	17.5 ± 1.0	16.6 ± 1.1	−0.68	2.42	0.741
	PLA	18.3 ± 0.6	17.6 ± 0.7	−0.24	1.65	
Semantic Fluency (Language)	ASI	17.7 ± 1.3	18.1 ± 1.22	−2.76	1.88	0.599
	PLA	17.3 ± 1.3	18.7 ± 0.95	−4.42	1.53	
Picture Naming (Language)	ASI	9.5 ± 0.5	10.5 ± 0.5	−2.54	0.54	0.236
	PLA	8.8 ± 0.3	9.8 ± 0.1	−1.48	−0.52	
Coding (Attention)	ASI	61.9 ± 2.5	63.3 ± 3.0	−5.24	2.49	0.176
	PLA	63.5 ± 2.5	61.2 ± 1.9	−1.25	−5.84	
Digit Span (Attention)	ASI	12.2 ± 0.6	12.3 ± 0.72	−1.55	1.43	0.578
	PLA	11.7 ± 0.4	12.4 ± 0.5	−1.81	0.25	
List Recall (DM)	ASI	7.3 ± 0.5	5.7 ± 0.8	0.46	2.79	0.438
	PLA	7.8 ± 0.4	6.9 ± 0.8	−0.86	2.64	
Story Recall (DM)	ASI	11.1 ± 0.6	10.9 ± 0.2	−11.29	11.19	0.347
	PLA	10.2 ± 0.4	10.3 ± 0.4	−1.23	1.73	
Figure Recall (DM)	ASI	18.3 ± 3.1	18.8 ± 0.3	−1.54	0.66	0.337
	PLA	17.8 ± 0.6	18.0 ± 0.5	−1.50	1.00	
List Recognition (DM)	ASI	19.6 ± 0.2	18.5 ± 0.5	0.03	2.22	0.560
	PLA	19.8 ± 0.1	18.8 ± 0.3	0.44	1.67	
Image Pair (Digital)	ASI	0.86 ± 0.04	0.91 ± 0.03	−0.09	0.01	0.480
	PLA	0.87 ± 0.04	0.93 ± 0.02	−0.10	−0.02	
Symbol Digit Match (Digital)	ASI	0.86 ± 0.04	0.92 ± 0.04	−0.14	0.03	0.546
	PLA	0.91 ± 0.04	0.92 ± 0.05	−0.09	0.07	

Note. means ± SE, ASI: Bonded Arginine Silicate, PLA: Placebo, CI = 95% confidence interval of the mean difference; IM = immediate memory, VSC = visuospatial/constructional; DM = delayed memory

## Data Availability

Data can be found in the Health, Human Performance, and Recreation department at the University of Arkansas in Fayetteville, AR, USA.
